# Author Correction: Performance assessment and economic analysis of a human Liver-Chip for predictive toxicology

**DOI:** 10.1038/s43856-023-00235-7

**Published:** 2023-01-12

**Authors:** Lorna Ewart, Athanasia Apostolou, Skyler A. Briggs, Christopher V. Carman, Jake T. Chaff, Anthony R. Heng, Sushma Jadalannagari, Jeshina Janardhanan, Kyung-Jin Jang, Sannidhi R. Joshipura, Mahika M. Kadam, Marianne Kanellias, Ville J. Kujala, Gauri Kulkarni, Christopher Y. Le, Carolina Lucchesi, Dimitris V. Manatakis, Kairav K. Maniar, Meaghan E. Quinn, Joseph S. Ravan, Ann Catherine Rizos, John F. K. Sauld, Josiah D. Sliz, William Tien-Street, Dennis Ramos Trinidad, James Velez, Max Wendell, Onyi Irrechukwu, Prathap Kumar Mahalingaiah, Donald E. Ingber, Jack W. Scannell, Daniel Levner

**Affiliations:** 1 Emulate Inc., 27 Drydock Avenue, Boston, MA USA; 2 Janssen Pharmaceuticals, Spring House, Philadelphia, PA USA; 3 Investigative Toxicology and Pathology, Abbvie, North Chicago, IL USA; 4 Wyss Institute for Biologically Inspired Engineering, Harvard University, Boston, MA USA; 5 Harvard John A. Paulson School of Engineering and Applied Sciences, Harvard University, Cambridge, MA USA; 6 Vascular Biology Program and Department of Surgery, Harvard Medical School and Boston Children’s Hospital, Boston, MA USA; 7 JW Scannell Analytics LTD, 32 Queens Crescent, Edinburgh, EH9 2BA UK

**Keywords:** Cell biology, Drug discovery

Correction to: *Communications Medicine* 10.1038/s43856-022-00209-1, published online 06 December 2022.

There was a mistake in Table 5 of this article. The specificity (%) value in the final row of Table 5 (the ‘Spheroid’ row) read ‘100’, but should have read ‘67’. There was also a typographical error in the Background section of the Abstract. ‘Organ-on-a-Chip technology has the potential improve success in drug development pipelines, as it can recapitulate organ-level pathophysiology and clinical responses’ should have read ‘Organ-on-a-Chip technology has the potential to improve success in drug development pipelines, as it can recapitulate organ-level pathophysiology and clinical responses’. Both mistakes have been corrected in the HTML and PDF versions of the article.

