# Author Correction: Performance assessment and economic analysis of a human Liver-Chip for predictive toxicology

**DOI:** 10.1038/s43856-023-00249-1

**Published:** 2023-02-02

**Authors:** Lorna Ewart, Athanasia Apostolou, Skyler A. Briggs, Christopher V. Carman, Jake T. Chaff, Anthony R. Heng, Sushma Jadalannagari, Jeshina Janardhanan, Kyung-Jin Jang, Sannidhi R. Joshipura, Mahika M. Kadam, Marianne Kanellias, Ville J. Kujala, Gauri Kulkarni, Christopher Y. Le, Carolina Lucchesi, Dimitris V. Manatakis, Kairav K. Maniar, Meaghan E. Quinn, Joseph S. Ravan, Ann Catherine Rizos, John F. K. Sauld, Josiah D. Sliz, William Tien-Street, Dennis Ramos Trinidad, James Velez, Max Wendell, Onyi Irrechukwu, Prathap Kumar Mahalingaiah, Donald E. Ingber, Jack W. Scannell, Daniel Levner

**Affiliations:** 1 Emulate Inc., 27 Drydock Avenue, Boston, MA USA; 2 Janssen Pharmaceuticals, Spring House, Philadelphia, PA USA; 3 Investigative Toxicology and Pathology, Abbvie, North Chicago, IL USA; 4 Wyss Institute for Biologically Inspired Engineering, Harvard University, Boston, MA USA; 5 Harvard John A. Paulson School of Engineering and Applied Sciences, Harvard University, Cambridge, MA USA; 6 Vascular Biology Program and Department of Surgery, Harvard Medical School and Boston Children’s Hospital, Boston, MA USA; 7 JW Scannell Analytics LTD, 32 Queens Crescent, Edinburgh, EH9 2BA UK

**Keywords:** Cell biology, Drug discovery

Correction to: *Communications Medicine* 10.1038/s43856-022-00209-1, published online 06 December 2022.

The data in Table 2 were erroneously duplicated in Table 3 of this article. The correct version of Table 3 is displayed below. Table 3 has been corrected in the HTML and PDF versions of this article.

**Table 3:** Results obtained with the expanded drug list.

Drug	Albumin IC_50_		ALT		Morphology		IF imaging	
	Donor 1	Donor 2	Donor 1	Donor 2	Donor 1	Donor 2	Donor 1	Donor 2
Asunaprevir	190	–	>1000	–	1000	–	Apoptosis	–
Benoxaprofen	8	20	>100	100	<0.1	100	Apoptosis	Apoptosis
Beta-Estradiol	>300	–	>300	–	>300	–	No findings	–
Chlorpheniramine	>300	–	>300	–	>300	–	No findings	–
Diclofenac	384	–	>1000	–	1000	–	Apoptosis	–
Labetalol	26	40	>100	100	>100	100	Apoptosis	Apoptosis
Lomitapide	4	–	>1000	–	<0.1	–	No findings	–
Simvastatin	<0.1	<10	>300	>300	>300	>300	No findings	Apoptosis
Stavudine	>60	132	300	>300	300	300	No findings	Mitotoxicity
Tacrine	76	>25	>300	>1000	>300	>1000	Apoptosis	Apoptosis
Telithromycin	13	–	30	–	100	–	Apoptosis	–
Ximelagatran	32	>300	30	>300	100	300	No findings	No findings
Zileuton	>100	210	>100	>300	>100	>300	No findings	No findings

