# Supplementary material for: Performance assessment and economic analysis of a human Liver-Chip for predictive toxicology
Source: Commun Med (Lond). 2022 Dec 6;2:154. doi: 10.1038/s43856-022-00209-1 (PMC9727064; doi:10.1038/s43856-022-00209-1)
Supplement: Supplementary file 9 — Supplementary information [file 43856_2022_209_MOESM9_ESM.pdf]

# Supplementary Information

Lorna Ewart[1*], Athanasia Apostolou[1], Skyler A. Briggs[1], Christopher V. Carman[1], Jake T. Chaff[1], Anthony R. Heng[1], Sushma Jadalannagari[1], Jeshina Janardhanan[1], Kyung-Jin Jang[1], Sannidhi R. Joshipura[1], Mahika M. Kadam[1], Marianne Kanellias[1], Ville J. Kujala[1], Gauri Kulkarni[1], Christopher Y. Le[1], Carolina Lucchesi[1], Dimitris V. Manatakis[1], Kairav K. Maniar[1], Meaghan E. Quinn[1], Joseph S. Ravan[1], Ann Catherine Rizos[1], John F.K. Sauld[1], Josiah D. Sliz[1], William Tien-Street[1], Dennis Ramos Trinidad[1], James Velez[1], Max Wendell[1], Onyi Irrechukwu[2], Prathap Kumar Mahalingaiah[3], Donald E. Ingber[4,5,6], Jack W. Scannell[7], Daniel Levner[1]

[1]Emulate Inc., 27 Drydock Avenue, Boston, MA, United States

[2]Janssen Pharmaceuticals, Spring House, Philadelphia, PA, United States

[3]Investigative Toxicology and Pathology, Abbvie, North Chicago, IL, United States

[4]Wyss Institute for Biologically Inspired Engineering, Harvard University, Boston, MA, United States

[5]Harvard John A. Paulson School of Engineering and Applied Sciences, Harvard University, Cambridge, MA, United States

[6]Vascular Biology Program and Department of Surgery, Harvard Medical School and Boston Children's Hospital, Boston, MA, United States

[7]JW Scannell Analytics LTD, 32 Queens Crescent, Edinburgh, EH9 2BA, United Kingdom

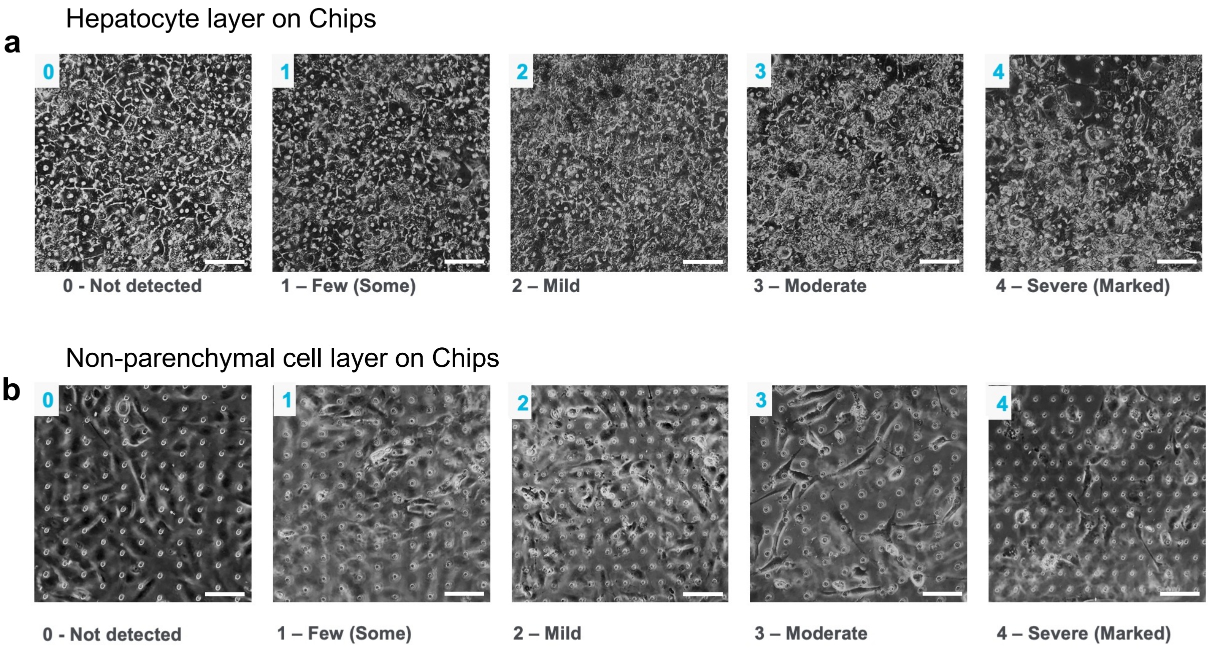


**Supplementary Figure 1. (a)** Representative brightfield images to depict the cellular morphology score in the top channel of the Liver-Chip which contains hepatocytes. A score of 0 represents no hepatotoxicity detected, which is defined by 95-100% healthy hepatocyte morphology, hexagonal shape containing binucleated cells, clear cell cytoplasm, distinctive cell junctions and less than 5% dead cells. A score of 1 represents at least 85% healthy hepatocyte morphology, a hexagonal shape containing binucleated cells, clear cell cytoplasm, distinctive cell junctions but < 15% are dead cells. A score of 2 represents mild hepatotoxicity with > 70% monolayer of hepatocytes visible, evidence that cells have begun to lose their distinct cell junctions, many cells contain a granulated cytoplasm but < 30% are dead cells. A score of 3 represents moderate hepatotoxicity with severe granulation of cytoplasm and most of the cells have lost their junctions. Approximately 50% of the cells are considered dead. A score of 4 represents severe hepatotoxicity with agglomeration of cell debris and > 50% of the cells are considered dead. The pores on the membrane become visible as there is no longer a cellular monolayer. **(b)** Representative brightfield images to depict the cellular morphology score in the bottom channel of the Liver-Chip which contains non-parenchymal cells. A score of 0 represents no cytotoxicity detected, with an intact monolayer and <1% of the cells are dead. A score of 1 represents at least 90% of the monolayer is present and there are <10% dead cells. A score of 2 represents mild cytotoxicity with > 80% of the monolayer present and < 20% are dead cells. A score of 3 represents moderate cytotoxicity with > 50% of the monolayer present and < 50% are dead cells. A score of 4 represents severe cytotoxicity with > 50% of the cells are considered dead. (bar, 50 µm).

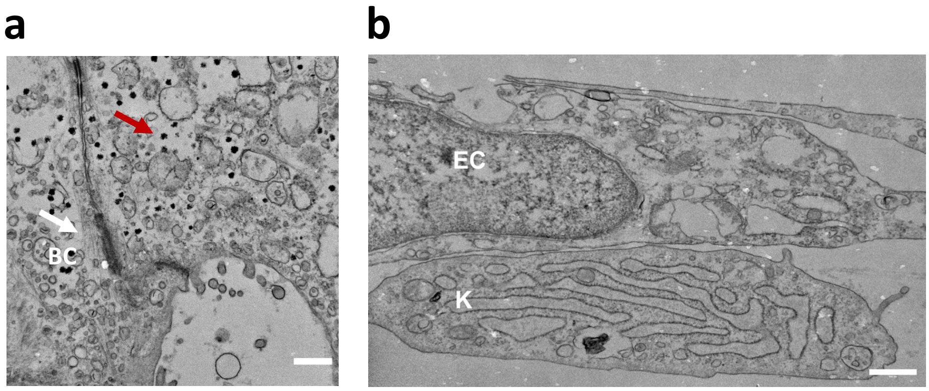


**Supplementary Figure 2.** Representative transmission electron microscopy images showing a well-formed bile canaliculus (bc) between neighboring hepatocytes **(a)** and cell-cell contact formation between a Kupffer (K) cell and liver sinusoidal endothelial cell **(b)** (bar, 0.5 μm).

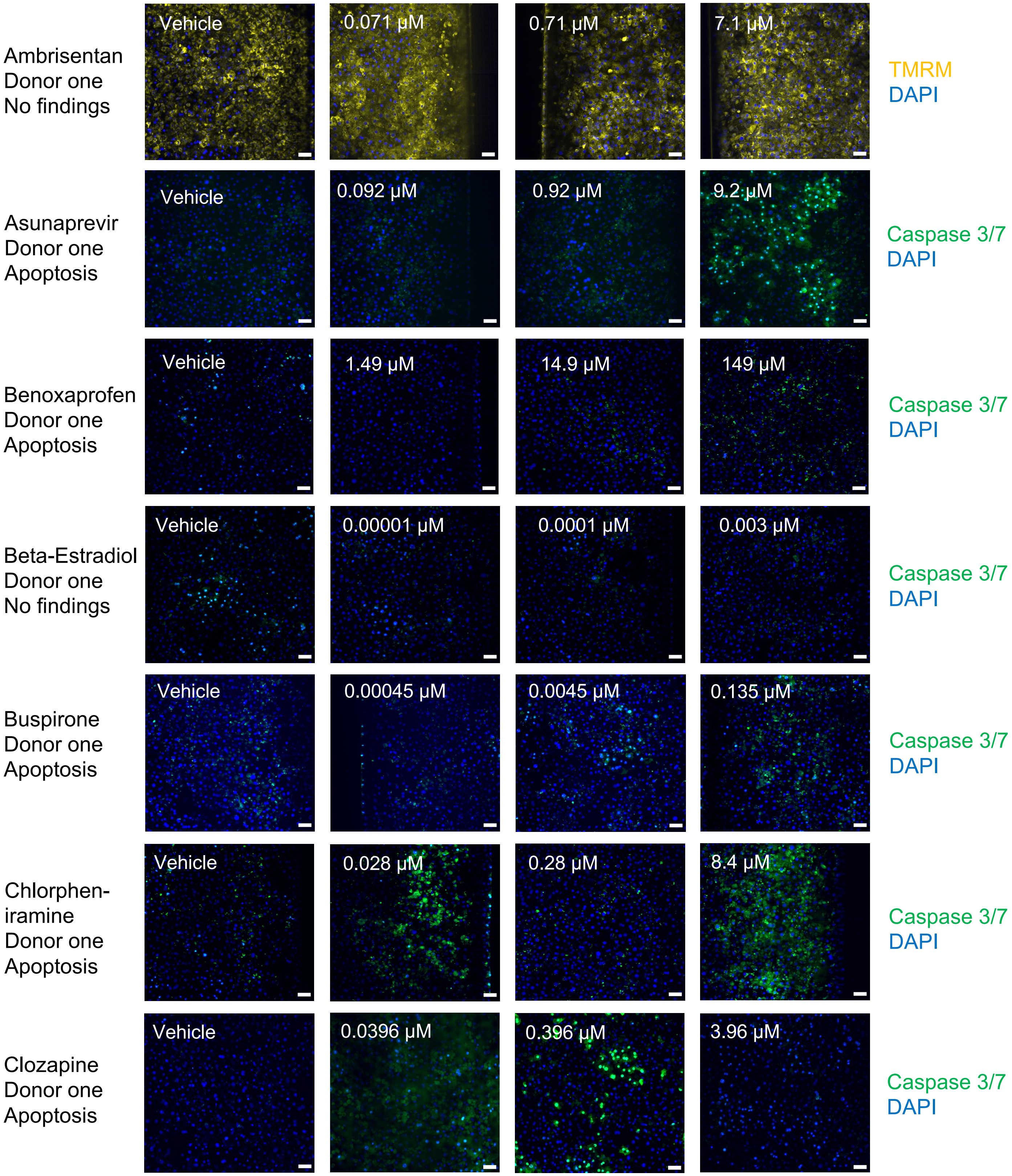


**Supplementary Figure 3.** Representative immunofluorescent images from day 7 post-vehicle or drug administration of the hepatocyte cell layer in the top channel of the chip. Each drug is shown with its free drug concentration and corresponding vehicle image that was used for thresholding across each donor the drug was tested in. The data support the immunofluorescent findings statement in **Tables 2 and 3**. (bar, 50 µm).

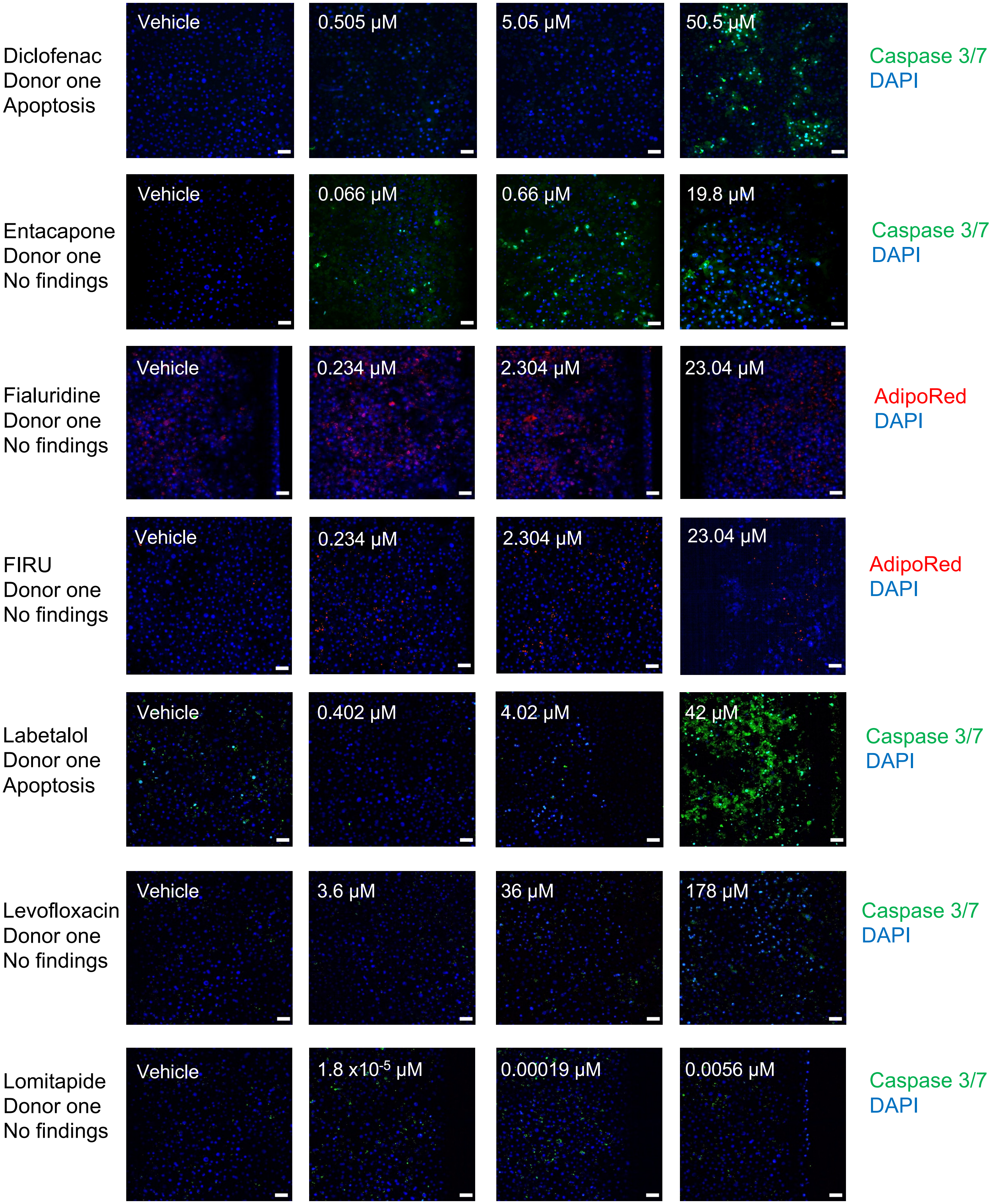


# Supplementary Figure 3

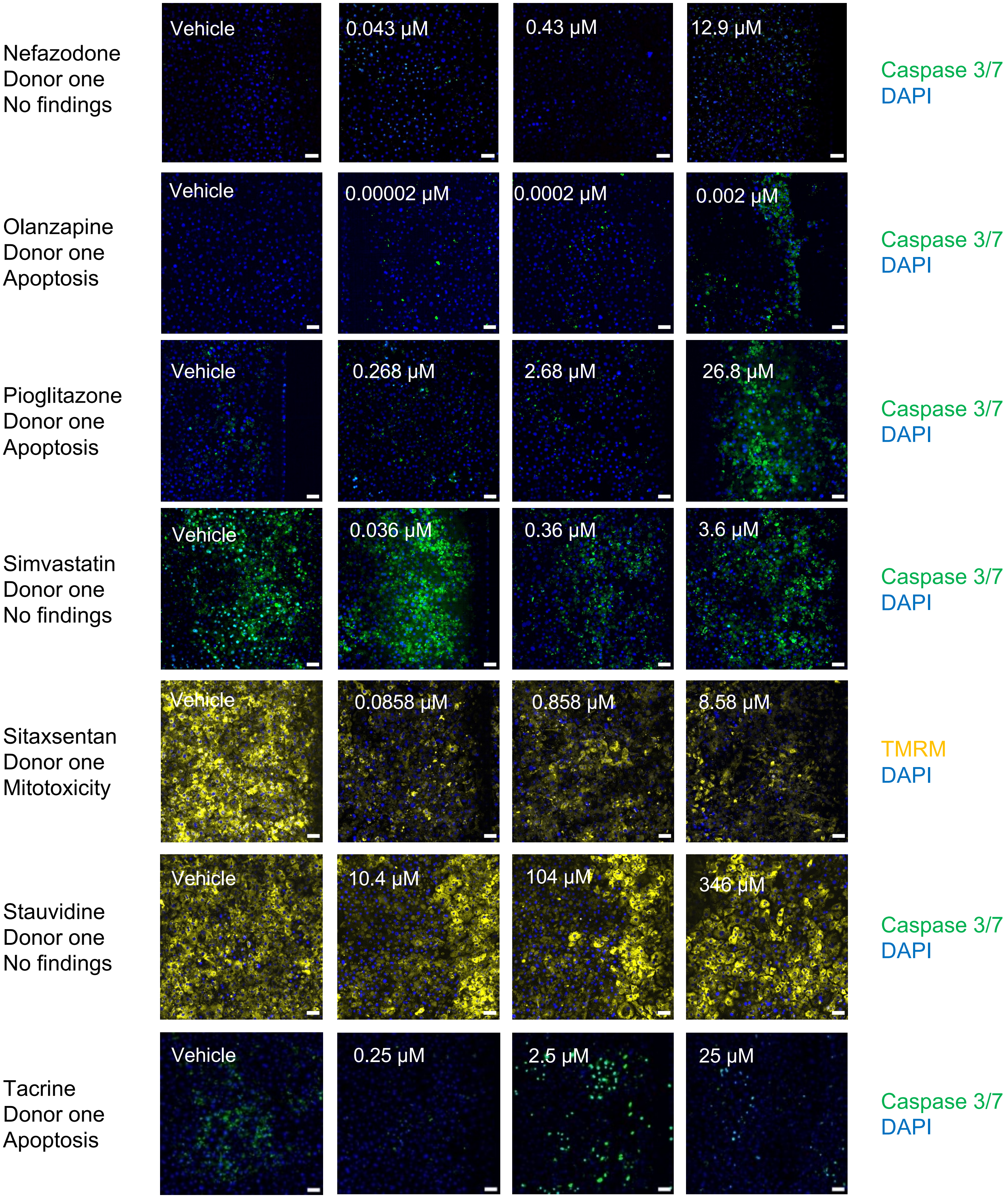


# Supplementary Figure 3

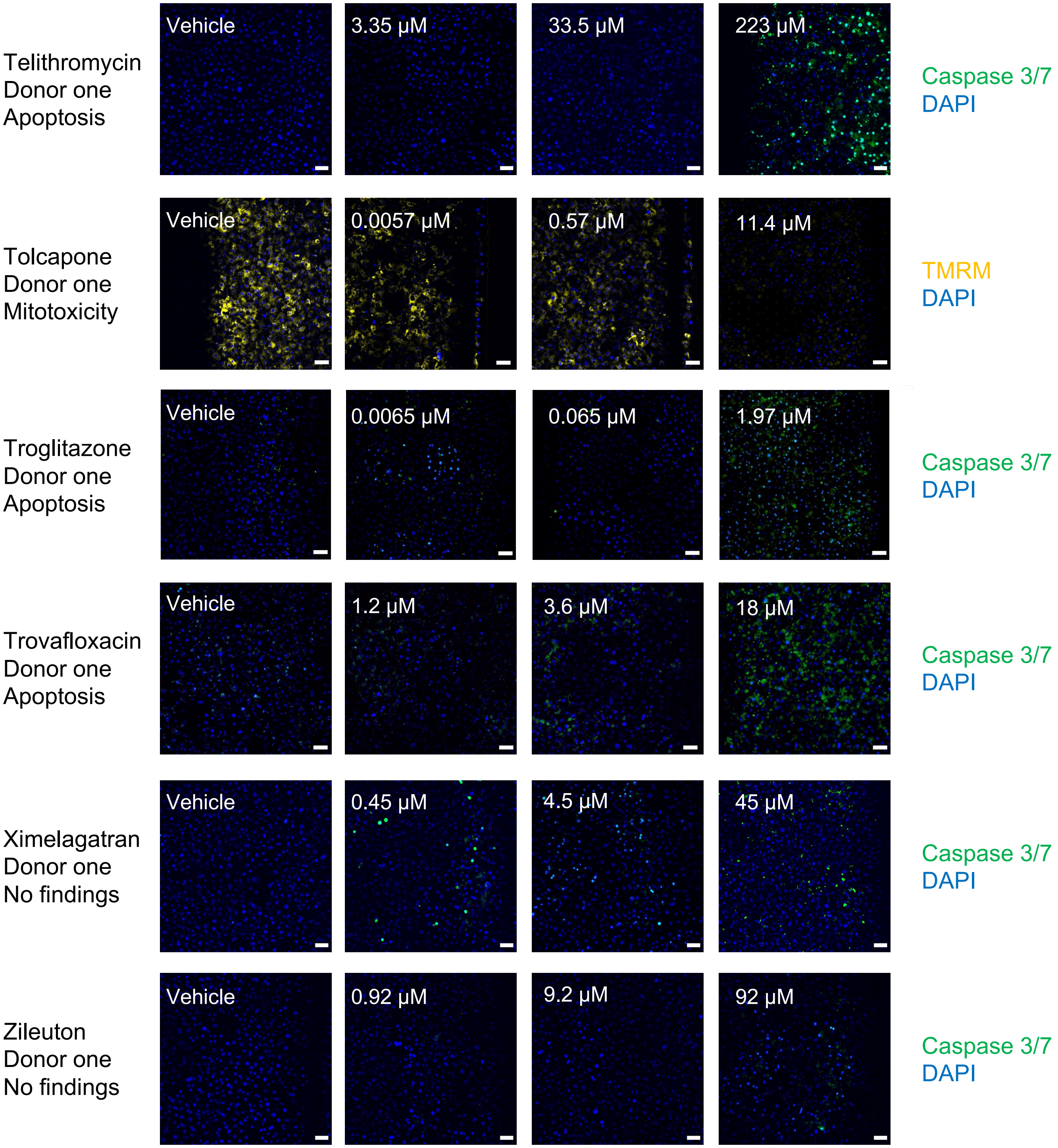


# Supplementary Figure 3

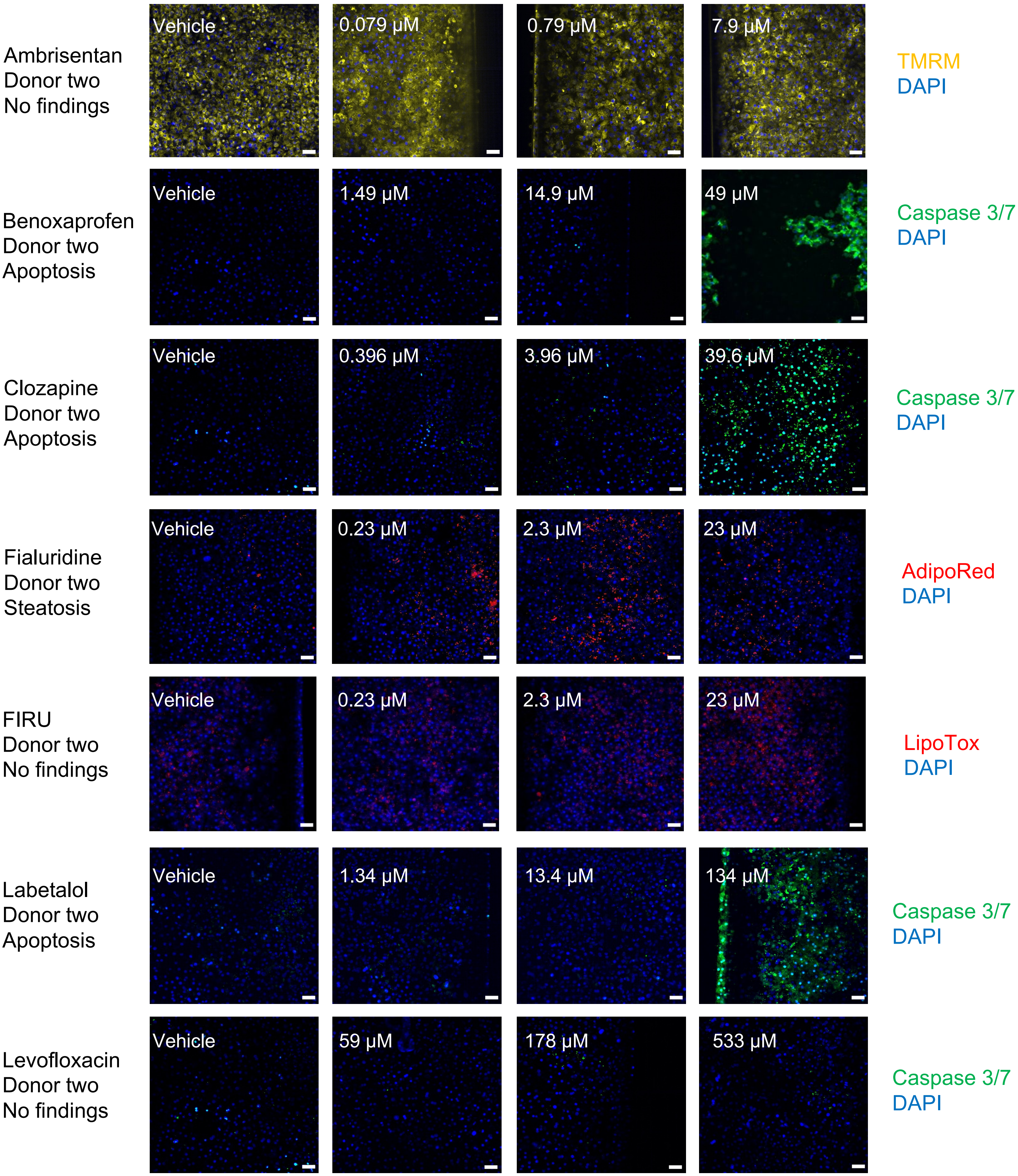


**Supplementary Figure 3**

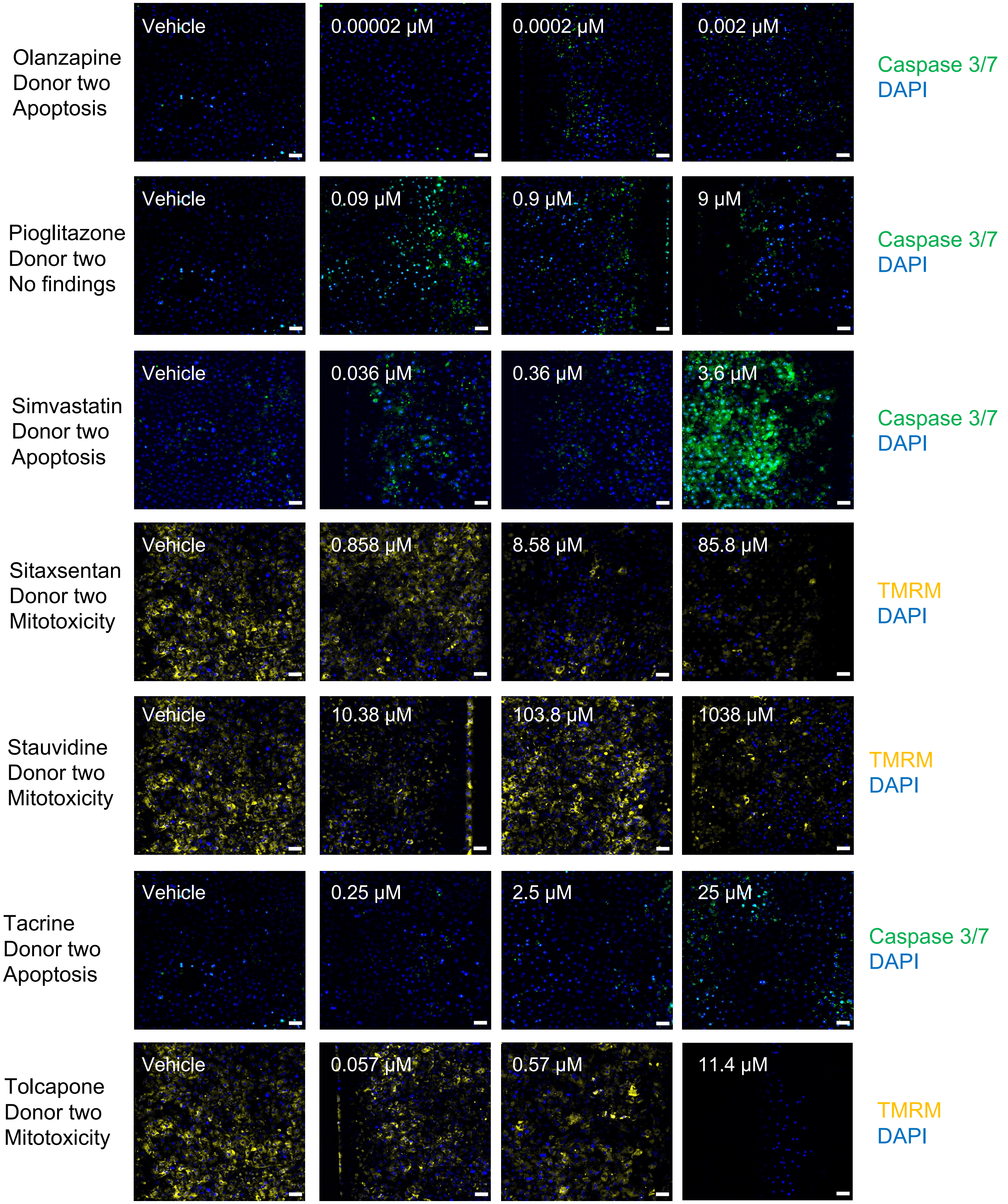


# Supplementary Figure 3

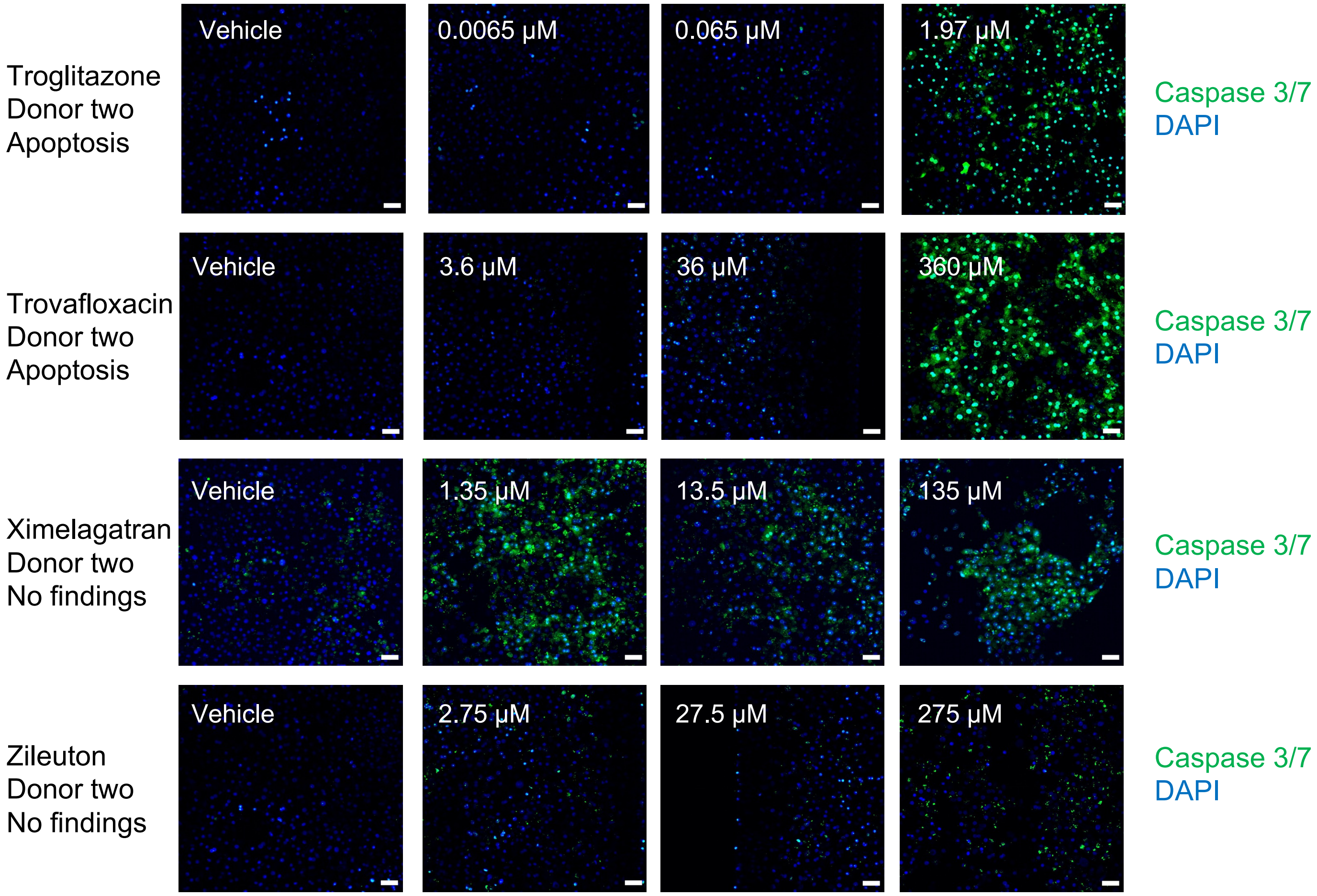


# Supplementary Figure 3

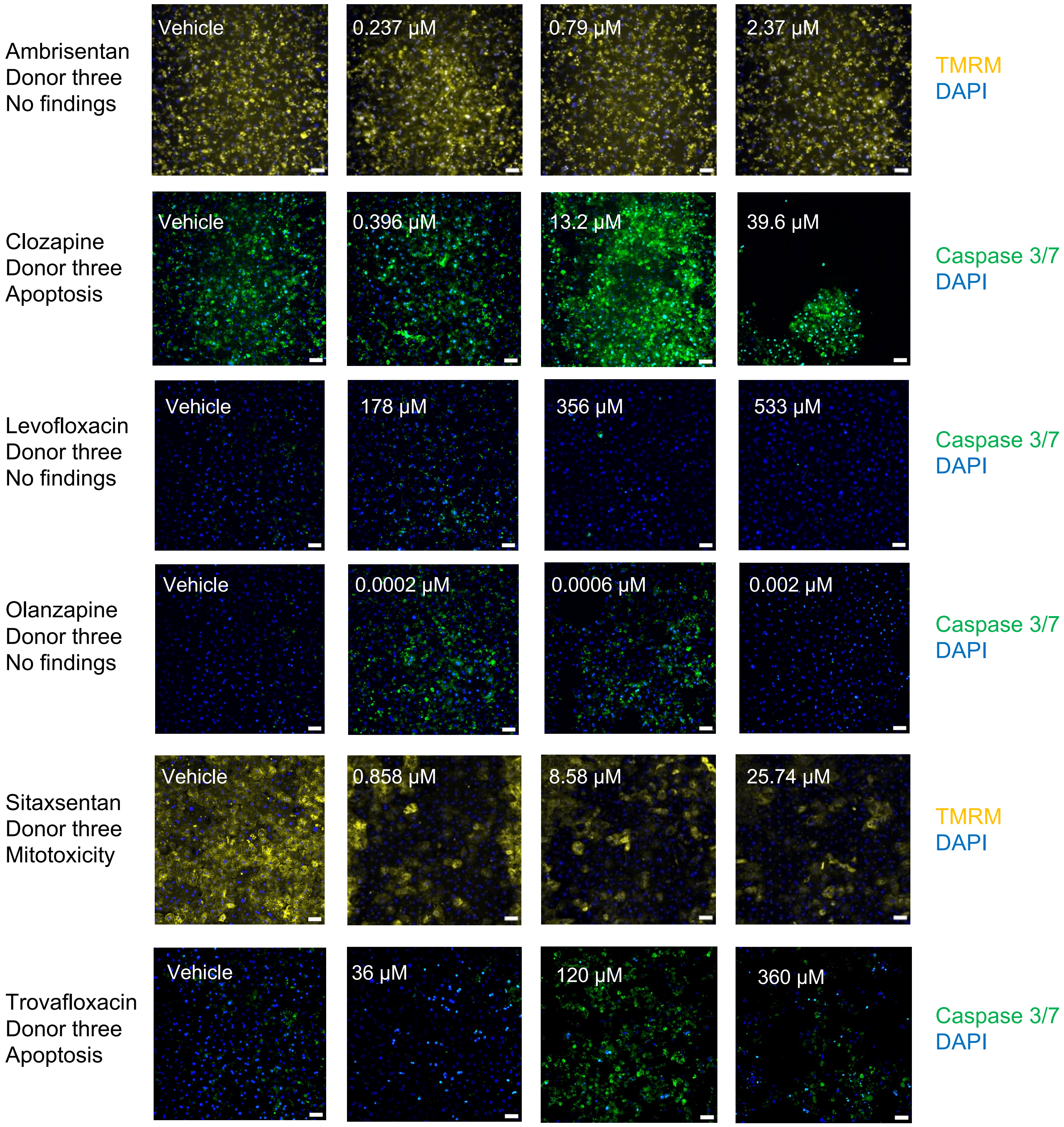


# Supplementary Figure 3

| Criteria/ Cell Type | Hepatocyte Donor one | Hepatocyte Donor two | Hepatocyte Donor three | Liver Sinusoidal Endothelial Cells | Kupffer Cells | Stellate Cells |
|---|---|---|---|---|---|---|
| Donor gender | Male | Male | Female | Male | Female | Male |
| Donor age | 53 years | 26 years | 77 years | Unknown | 25 years | 21-25 years |
| Race | Caucasian | Caucasian | African-American | Unknown | Caucasian | Unknown |
| BMI | 18.1 | 22.2 | 28.5 | Unknown | 19.7 | 20-25 |
| Viability | 90% | 93% | 96% | 93% | 70% | 91% |
| Phenotypic markers | MRP2 positive Vimentin negative | MRP2 positive Vimentin negative | MRP2 positive | Stabilin positive Vimentin positive | CD68 positive CD11b positive Alpha-SMA negative | Vimentin positive AdipoRed positive Alpha SMA positive |
| Vendor | Thermo | Thermo | Thermo | Cell Systems | Lonza | iXCells |
| Product Codes/ Catalog Numbers | HMCPTS | HMCPTS | HMCPTS | ACBRI 566 | HLKC | 10HU-210 |
| Lot | HU8305 | HU8350 | HU2005 | 566.03.03.05.0M | HL1800080KC | 300075-5 |
| CoA Available Online | Yes | Yes | Yes | No | No | No |

**Supplementary Table 1.** Details of the cell sources and their defining characteristics used in the investigation.

| Incremental DILI sensitivity | Tox FNR vs. Baseline | Clinical dev. Success rate | Portfolio IRR | Portfolio eNPV ($m) | NPV of chip costs ($m) | IRR on chip investment | Portfolio NPV / NPV of base R&D costs | Steady state uplift in pre-tax profit (%) | Annualized industry-wide pre-tax profit uplift ($m) |
|---|---|---|---|---|---|---|---|---|---|
| 0.0% | 100.0% | 11.5% | 11.0% | -3.5 | -3.5 | -3.5% | -0.2% | -0.1% | (207) |
| 7.5% | 99.0% | 11.5% | 11.0% | 1.1 | -3.6 | 14.4% | 0.1% | 0.0% | 61 |
| 14.9% | 98.0% | 11.6% | 11.0% | 5.7 | -3.6 | 24.7% | 0.3% | 0.2% | 331 |
| 22.4% | 97.0% | 11.6% | 11.1% | 10.3 | -3.6 | 30.4% | 0.5% | 0.3% | 602 |
| 29.8% | 96.0% | 11.7% | 11.1% | 15.0 | -3.6 | 33.8% | 0.8% | 0.5% | 876 |
| 37.3% | 95.0% | 11.7% | 11.2% | 19.8 | -3.6 | 36.0% | 1.0% | 0.7% | 1,151 |
| 44.7% | 94.0% | 11.7% | 11.2% | 24.5 | -3.6 | 37.6% | 1.3% | 0.8% | 1,428 |
| 52.2% | 93.0% | 11.8% | 11.2% | 29.3 | -3.6 | 38.7% | 1.6% | 1.0% | 1,707 |
| 61.5% | 91.8% | 11.8% | 11.3% | 35.3 | -3.6 | 39.9% | 1.9% | 1.2% | 2,058 |
| 67.1% | 91.0% | 11.9% | 11.3% | 39.0 | -3.7 | 40.4% | 2.1% | 1.3% | 2,270 |
| 74.5% | 90.0% | 11.9% | 11.4% | 43.9 | -3.7 | 41.0% | 2.3% | 1.5% | 2,555 |
| 82.0% | 89.0% | 11.9% | 11.4% | 48.8 | -3.7 | 41.5% | 2.6% | 1.6% | 2,841 |
| 87.0% | 88.3% | 12.0% | 11.4% | 52.1% | -3.7 | 41.8% | 2.8% | 1.8% | 3,034 |
| 96.0% | 87.1% | 12.0% | 11.5% | 58.1% | -3.7 | 42.3% | 3.1% | 2.0% | 3,384 |

**Supplementary Table 2.** Portfolio value and industry profits increase with reductions in the false negative rate (FNR) of the preclinical toxicology assessment, which cause fewer toxic drugs to enter the clinic. The leftmost column tabulates the proportional improvement in DILI detection versus the base case, and the next column shows the improvement in the toxicology FNR relative to the model's base case. Since DILI is around 13% or tox failure in development, near total DILI sensitivity can reduce the FNR to around 87% of its base case value. Subsequent columns then show the clinical development success rate from entry into Phase I to launch, the internal rate of return (IRR) of the R&D portfolio, the NPV of the portfolio discounted to the time of drug launch, the capitalized cost of Liver-Chips used in assessing the portfolio (discounted to the time of launch), and the marginal IRR on the Liver-Chip investment. The remaining columns capture the value uplift due to FNR improvement as a percentage uplift of the portfolio's NPV relative to the baseline NPV of R&D, percentage uplift of steady state pre-tax profits, and the estimated increase in annual pre-tax profits for the small-molecule drug development industry. The row highlighted in dark gray relates to the improvement in FNR that may result from incorporating the Liver-Chip into DILI prediction workflows in accordance with the 87% sensitivity estimated by the present study. The rows highlighted in light gray correspond to the 95% confidence interval around this point estimate. The economic model behind these calculations is provided in full in the Supplementary Materials (Supplementary Data 2).
