# Supplementary material for: Performance assessment and economic analysis of a human Liver-Chip for predictive toxicology
Source: Commun Med (Lond). 2022 Dec 6;2:154. doi: 10.1038/s43856-022-00209-1 (PMC9727064; doi:10.1038/s43856-022-00209-1)
Supplement: Supplementary file 11 — Description of Additional Supplementary Files [file 43856_2022_209_MOESM11_ESM.pdf]

Description of Additional Supplementary Files

File Name: Supplementary Data 1

Description: This is a summary of each drug used in each cycle of the study. Reference data was obtained from the literature, and this is reflected in the table.

File Name: Supplementary Data 2

Description: additional information about: (i) Analytic derivation of base case, (ii) Attrition parameter details, (iii) Pipeline and financial model, (iv) DILI and tox sensitivity analyses, (v) NPV to annualized financial value, (vi) References, (vii) Supplemental details on the method, (viii) Source for Supplemental results figure and (ix) Discussion of broad potential of improved predictive toxicology.

File Name: Supplementary Data 3

Description: The albumin concentration levels presented in Figure 2g

File Name: Supplementary Data 4

Description: The urea concentration levels presented in Figure 2h

File Name: Supplementary Data 5

Description: The statistical significance results for Albumin and Urea concentration levels across different donors and days (Figure 2g and 2h)

File Name: Supplementary Data 6

Description: The statistical significance results of the gene expression levels between Day 3 and Day 7 for donors 2 and 3 (Figure 2i-2j)

File Name: Supplementary Data 7

Description: The gene expression levels in TPM presented in Figure 2i-2j for donors 2 and 3

File Name: Supplementary Data 8

Description: Albumin, ALT and morphological injury score data presented in Figure 1a-1i for clozapine and olanzapine, troglitazone and pioglitazone, and trovafloxacin and levofloxacin
