# Supplementary material for: Performance assessment and economic analysis of a human Liver-Chip for predictive toxicology
Source: Commun Med (Lond). 2022 Dec 6;2:154. doi: 10.1038/s43856-022-00209-1 (PMC9727064; doi:10.1038/s43856-022-00209-1)
Supplement: Supplementary file 12 — Reporting Summary [file 43856_2022_209_MOESM12_ESM.pdf]

nature research

Corresponding author(s): Lorna Ewart
Last updated by author(s): Oct 19, 2022

Reporting Summary

Nature Research wishes to improve the reproducibility of the work that we publish. This form provides structure for consistency and transparency in reporting. For further information on Nature Research policies, see our Editorial Policies and the Editorial Policy Checklist.

Statistics

For all statistical analyses, confirm that the following items are present in the figure legend, table legend, main text, or Methods section.

n/a	Confirmed	
☐	☒	The exact sample size (*n*) for each experimental group/condition, given as a discrete number and unit of measurement
☐	☒	A statement on whether measurements were taken from distinct samples or whether the same sample was measured repeatedly
☐	☒	The statistical test(s) used AND whether they are one- or two-sided<br>*Only common tests should be described solely by name; describe more complex techniques in the Methods section.*
☒	☐	A description of all covariates tested
☒	☐	A description of any assumptions or corrections, such as tests of normality and adjustment for multiple comparisons
☐	☒	A full description of the statistical parameters including central tendency (e.g. means) or other basic estimates (e.g. regression coefficient) AND variation (e.g. standard deviation) or associated estimates of uncertainty (e.g. confidence intervals)
☐	☒	For null hypothesis testing, the test statistic (e.g. *F*, *t*, *r*) with confidence intervals, effect sizes, degrees of freedom and *P* value noted<br>*Give P values as exact values whenever suitable.*
☒	☐	For Bayesian analysis, information on the choice of priors and Markov chain Monte Carlo settings
☒	☐	For hierarchical and complex designs, identification of the appropriate level for tests and full reporting of outcomes
☐	☒	Estimates of effect sizes (e.g. Cohen's *d*, Pearson's *r*), indicating how they were calculated

Our web collection on statistics for biologists contains articles on many of the points above.

Software and code

Policy information about availability of computer code

Data collection	Microscopy: ECHO - Revolve, Opera Phenix (Perkin Elmer), Zeiss Zen 2.3<br>Transcriptomic data: Illumina HiSeq 4000, 2x150 PE system
Data analysis	1. Statistical analysis - Prism 8, RStudio Version 1.3.1056, packages, matrixStats, drc, sandwich, lmtest, ggplot2, multcomp, tidyr, scales<br>2. RNA-Sequencing, Trimmomatic v.0.36, STAR aligner v.2.5.2b, Homo Sapiens GRCh38, Subread package v.1.5.2 featureCounts<br>3. Harmony imaging and analysis software<br>4. ImageJ with Java 1.8.0_172

For manuscripts utilizing custom algorithms or software that are central to the research but not yet described in published literature, software must be made available to editors and reviewers. We strongly encourage code deposition in a community repository (e.g. GitHub). See the Nature Research guidelines for submitting code & software for further information.

Data

Policy information about availability of data

All manuscripts must include a data availability statement. This statement should provide the following information, where applicable:

- Accession codes, unique identifiers, or web links for publicly available datasets
- A list of figures that have associated raw data
- A description of any restrictions on data availability

In the Manuscript the following is stated:
All data generated or analyzed during this study are included in this published article (and its supplementary information files) or are available from the corresponding authors on reasonable request. RNA sequencing data have been deposited in the National Center for Biotechnology Information Gene Expression Omnibus (GEO) under accession number GSE207339.

Field-specific reporting

Please select the one below that is the best fit for your research. If you are not sure, read the appropriate sections before making your selection.

☒ Life sciences ☐ Behavioural & social sciences ☐ Ecological, evolutionary & environmental sciences

For a reference copy of the document with all sections, see nature.com/documents/nr-reporting-summary-flat.pdf

Life sciences study design

All studies must disclose on these points even when the disclosure is negative.

Sample size	To determine the sample size, we used data from previous experiments to generate large synthetic datasets under different noise levels. These datasets, were used to estimate for each condition the "optimal" (subject to RMSE) minimum sample size for the expected level of noise in the data.
Data exclusions	Data was not excluded from the experiment unless apparent failures due to following reasons:<br>1-Hardware failure (e.g. flow issues)<br>2-Contamination problems
Replication	All experiments were replicated with at least 2 Chip set-ups.
Randomization	Samples were allocated into experimental groups at random.
Blinding	The investigators were not blind to the design of the study; however compound treatment was carried out blinded by the operators

Reporting for specific materials, systems and methods

We require information from authors about some types of materials, experimental systems and methods used in many studies. Here, indicate whether each material, system or method listed is relevant to your study. If you are not sure if a list item applies to your research, read the appropriate section before selecting a response.

Materials & experimental systems

n/a	Involved in the study
☒	☐ Antibodies
☒	☐ Eukaryotic cell lines
☒	☐ Palaeontology and archaeology
☒	☐ Animals and other organisms
☒	☐ Human research participants
☒	☐ Clinical data
☒	☐ Dual use research of concern

Methods

n/a	Involved in the study
☒	☐ ChIP-seq
☒	☐ Flow cytometry
☒	☐ MRI-based neuroimaging
